# Longitudinal Numbers-Needed-To-Treat (NNT) for Achieving Various Levels of Analgesic Response and Improvement with Etoricoxib, Naproxen, and Placebo in Ankylosing Spondylitis

**DOI:** 10.1186/1471-2474-12-165

**Published:** 2011-07-18

**Authors:** Paul M Peloso, Arnold Gammaitoni, Steven S Smugar, Hongwei Wang, Andrew R Moore

**Affiliations:** 1 Clinical Research, Merck Sharp & Dohme Corp., 1 Merck Drive, Whitehouse Station, NJ, 08889, USA; 2 Global Center for Scientific Affairs. Merck Sharp & Dohme Corp., 1 Merck Drive, Whitehouse Station, NJ, 08889, USA; 3 Global Scientific & Medical Publications, Merck Sharp & Dohme Corp., 1 Merck Drive, Whitehouse Station, NJ, 08889, USA; 4 Biostatistics & Research Decision Sciences, Merck Sharp & Dohme Corp., 1 Merck Drive, Whitehouse Station, NJ, 08889, USA; 5 Pain Research and Nuffield Department of Anaesthetics, The Churchillville Hospital, University of Oxford, Oxford, OX3 7LJ, UK

## Abstract

**Background:**

Clinical analgesic trials typically report response as group mean results. However, research has shown that few patients are average and most have responses at the extremes. Moreover, group mean results do not convey response levels and thus have limited value in representing the benefit-risk at an individual level. Responder analyses and numbers-needed-to-treat (NNT) are considered more relevant for evaluating treatment response. We evaluated levels of analgesic response and Bath Ankylosing Spondylitis Disease Activity Index (BASDAI) score improvement and the associated NNTs.

**Methods:**

This was a post-hoc analysis of a 6-week, randomized, double-blind study (N = 387) comparing etoricoxib 90 mg, etoricoxib 120 mg, naproxen 1000 mg, and placebo in AS. Spine pain and BASDAI were measured on a 100-mm visual analog scale. The number and percentage of patients achieving ≥30% and ≥50% improvement in both BASDAI and spine pain were calculated and used to determine the corresponding NNTs. Patients who discontinued from the study for any reason were assigned zero improvement beyond 7 days of the time of discontinuation.

**Results:**

For etoricoxib 90 mg, etoricoxib 120 mg and naproxen 1000 mg, the NNTs at 6 weeks compared with placebo were 2.0, 2.0, and 2.7 respectively for BASDAI ≥30% improvement, and 3.2, 2.8, and 4.1 for ≥50% improvement. For spine pain, the NNTs were 1.9, 2.0, and 3.2, respectively, for ≥30% improvement, and 2.7, 2.5, and 3.7 for ≥50% improvement. The differences between etoricoxib and naproxen exceeded the limit of ±0.5 units described as a clinically meaningful difference for pain. Response rates and NNTs were generally similar and stable over 2, 4, and 6 weeks.

**Conclusions:**

For every 2 patients treated with etoricoxib, 1 achieved a clinically meaningful (≥30%) improvement in spine pain and BASDAI beyond that expected from placebo, whereas the corresponding values were approximately 1 in every 3 patients treated with naproxen. Use of NNTs and responder analyses provide additional, complementary information beyond population mean responses when assessing efficacy compared to placebo and amongst active therapies.

## Background

Clinical trials of analgesic medications in ankylosing spondylitis (AS) and other disease states typically report response as group mean results. However, research has shown that few patients are average and most have analgesic responses at the extremes [1-4]. For example, we have previously shown that in chronic low back pain, patient assessment of response to treatment has more of a U-shaped curve rather than a bell-shaped curve [4]. While it is implied that the group mean is applicable to most patients, it actually applies to few patients. Moreover, by assuming a bell-shaped curve for responders, group means imply ranges and distributions for responses that are not correct when viewed from the perspective of individual patient responses, and therefore are of lesser value for communicating treatment expectations with patients.

A more practical measure to estimate response is an individual patient responder analysis, which calculates the distribution of the magnitude of responses across individual patients, as has been done for osteoarthritis [5] and fibromyalgia [6]. Such an approach provides the proportion of patients achieving specified response levels, and thus adds the additional dimension of a clinically important change in patient response expected of a given therapy. Since adverse events are typically presented as rates, use of responder analyses puts both benefit and risk on the same metric and further facilitates benefit-risk discussions with individual patients and can help clinicians make more informed decisions.

The numbers-needed-to-treat (NNT) approach allows a comparison to placebo in a single number, and conveys additional information about expected treatment benefits, beyond that expected for with placebo in a straightforward manner. It has been suggested that a difference of ± 0.5 NNT units is a clinically meaningful difference [7-10].

The Initiative on Methods, Measurement, and Pain Assessment in Clinical Trials (IMMPACT) group suggested that responder analyses in pain should be based on established thresholds of change considered to be important [7]. Specifically, they suggest that the minimal clinically important difference in improvement is a 10-20% decrease on a 0-to-10 numerical rating scale, whereas improvements of ≥30% and ≥50% represent moderately important and substantial improvements [7]. An additional improvement of ≥70% is considered "extensive" [4-6]. Similarly, it has been demonstrated that the minimal clinically important difference in the Bath Ankylosing Spondylitis Disease Activity Index (BASDAI), an outcome measure specific to AS, has been reported to be a 22% improvement [11]. Thus changes of 30%, 50% and 70% on the BASDAI are of certain clinical benefit.

The purpose of this post-hoc analysis was to evaluate the proportions of patients achieving improvements from baseline in BASDAI and spine pain of 15%, 30%, 50%, and 70% and their associated NNTs over time in a study of etoricoxib, naproxen, and placebo in AS [12]. Specifically, we sought to describe the influence of various time points as well as various thresholds of response, on the responder proportions and associated NNTs, in an AS population.

## Methods

### Study design and patients

The methods of the trial have been previously published in detail [12]. Briefly, this was a double-blind, parallel-group, 52-week active-comparator and placebo-controlled study comparing etoricoxib 90 mg, etoricoxib 120 mg, and naproxen 1000 mg. The study consisted of a 6-week placebo-controlled period (Part I), and an optional 46-week, active-comparator period (Part II). Patients who received placebo in Part I were assigned to one of the 3 active treatment groups in equal ratios. Patients who received active treatment in Part I remained on that treatment for Part II. The original published study on which this secondary analysis is based was conducted in accordance with the standards established by the Declaration of Helsinki. For each study site, the protocol and consent form were approved by an institutional review board committee or ethics review committee. All patients provided written informed consent before enrollment.

Patients were ≥18 years of age, had a diagnosis of AS based on the modified New York criteria for AS [13] ≥6 months prior to study start, a history of therapeutic benefit with nonsteroidal anti-inflammatory drugs (NSAIDs), and routine NSAID use at a therapeutic dose level for ≥30 days prior to enrollment. Patients were also required to have used approved nonstudy antirheumatic therapy (e.g., methotrexate, sulfasalazine) at stable dose. After washout of prestudy NSAIDs, patients had to demonstrate a worsening of spine pain of ≥40 mm on a 100-mm visual analog scale (VAS), and an increase of ≥30% (minimum 12 mm) compared with the rating at the screening visit. Pertinent exclusion criteria included concurrent rheumatic disease (e.g., systemic lupus erythematosus, gout), but patients with chronic peripheral arthritis were eligible if spine pain was the primary source of pain. Excluded medications included corticosteroids within 1 month of screening, or analgesics within 3 days of study entry and throughout the study. Low-dose aspirin (≤100 mg once daily) was permitted for cardiovascular prophylaxis, and acetaminophen was available for rescue analgesia.

Clinical assessments were performed at screening, flare/randomization, and at weeks 2, 4, 6, 8, 16, 26, 43, and 52, and/or at the discontinuation visit. The co-primary endpoints of the original study were the patient assessment of spinal pain (100-mm VAS), patient global assessment of disease activity (100-mm VAS), and the Bath Ankylosing Spondylitis Functional Index (100-mm VAS). Secondary endpoints included patient global assessment of response to therapy (PGART; 0 = excellent to 4 = no response) and BASDAI (100-mm VAS).

### Responder analysis

The present responder analysis was limited to the initial 6-week placebo-controlled portion of the trial, and followed principles from IMMPACT [7] and ACTINPAIN [14] regarding outcomes, imputation methods, and evidence quality. The placebo portion of the trial was limited to 6-weeks due to practical and ethical limitations of denying AS patients in pain access to effective therapy.

The PGART is a straightforward assessment of response, on a simple 5-point scale. For each treatment arm we calculated the number and percentage patients at each PGART level at Week 6. For both BASDAI and spine pain, the number and percentage of patients achieving ≥15%, ≥30%, ≥50%, and ≥70% improvement from baseline were calculated at Weeks 2, 4, and 6 and these values were used to determine the corresponding NNTs. Patients who discontinued from the study for any reason were assigned zero improvement beyond 7 days from the time of discontinuation.

Since this exploratory analysis was descriptive in nature, no formal statistical hypothesis testing was performed, nor was adjustment made for multiplicity.

## Results

### Patients

Baseline patient characteristics are shown in Table 1. Most patients were male, had a mean age of 44 years, a mean baseline spine pain of 77 mm, and a mean BASDAI score of 55 mm.

**Table 1:** Baseline patient characteristics

	**Placebo N = 93**	**Etoricoxib 90 mg N = 103**	**Etoricoxib 120 mg N = 92**	**Naproxen 1000 mg N = 99**
**Age (years), mean (SD)**	43.7 (12.1)	43.1 (12.1)	42.5 (12.0)	45.0 (11.4)
**Female (%)**	20.4	26.2	21.7	20.2
**History of iritis (%)**	33.3	37.9	31.5	31.3
**History of chronic peripheral arthritis (%)**	39.8	39.8	39.1	41.4
**History of corticosteroid use (%)**	32.3	23.3	23.9	22.2
Concomitant DMARD use (%)*	19.4	26.2	19.6	23.2
**Baseline BASDAI (100-mm VAS), mean (SD)**	54.1 (27.0)	56.9 (22.5)	55.2 (25.1)	54.1 (23.2)
**Baseline spine pain (100-mm VAS), mean (SD)**	77.2 (15.2)	78.0 (13.9)	78.0 (14.2)	77.2 (16.5)

*Auranofin, azathioprine, cyclosporine, leflunomide, methotrexate, methotrexate sodium, or sulfasalazine

DMARD: disease-modifying antirheumatic drug; SD: standard deviation; VAS: visual analog scale

### PGART

**Figure 1:**
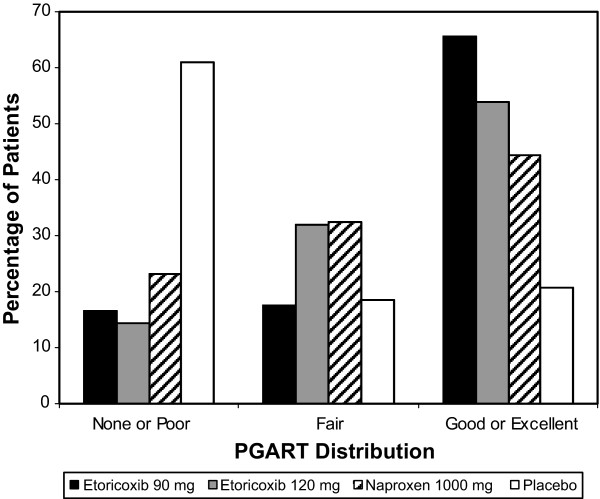
Distribution of PGART response at 6 weeks.

As shown in Figure 1, the distribution of PGART responses at 6 weeks for the individual treatment groups does not show a normal, Gaussian distribution

### Responders

**Figure 2:**
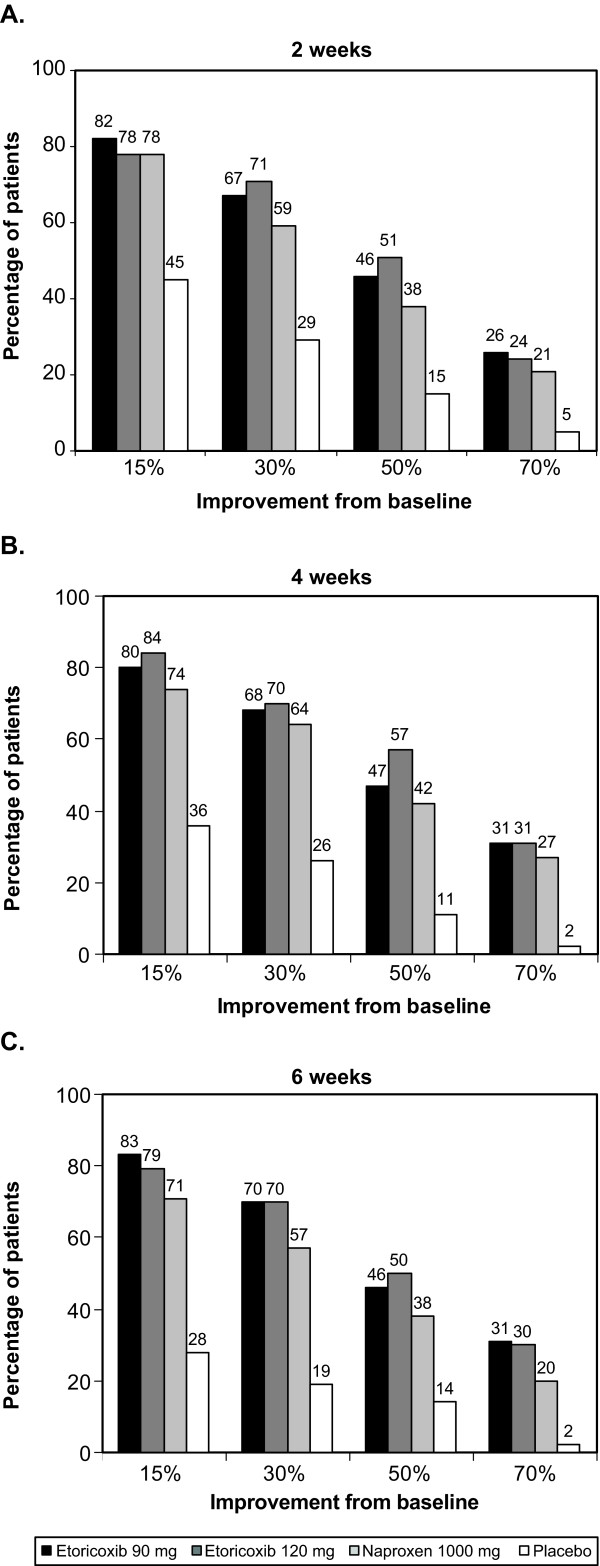
**Proportions of patients achieving various thresholds of improvement in BASDAI from baseline at (A) 2 weeks, (B), 4 weeks, and (C) 6 weeks**.

**Figure 3:**
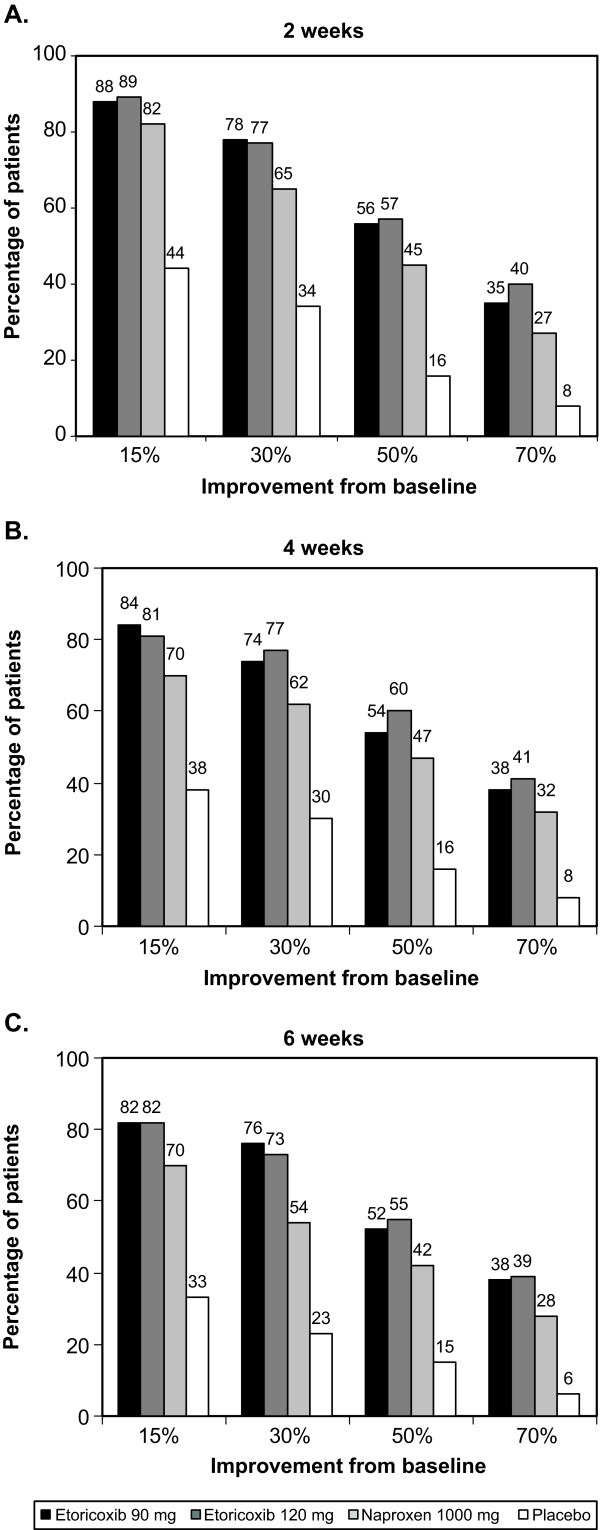
**Proportions of patients achieving various thresholds of improvement in spine pain from baseline at (A) 2 weeks, (B) 4 weeks, and (C) 6 weeks**.

Predictably, for both BASDAI (Figure 2) and spine pain (Figure 3), a greater proportion of patients achieved a 15% improvement from baseline than a 70% improvement from baseline with active drug and placebo. The proportions of patients achieving various thresholds of response were generally stable over Weeks 2 through 6 for patients receiving active treatment. Response with placebo was highly dependent on the level of response examined, falling at six weeks from about 30% to 5% or below for both outcomes. There was also a tendency for placebo response rates to fall over the 2-6 week time frame. For all thresholds of response, substantially more patients receiving active treatment achieved a given threshold compared to those receiving placebo, and in most cases, numerically more patients receiving etoricoxib 90 mg or 120 mg achieved a given level of response compared to those receiving naproxen 1000 mg.

### Numbers-Needed-to-Treat

**Figure 4:**
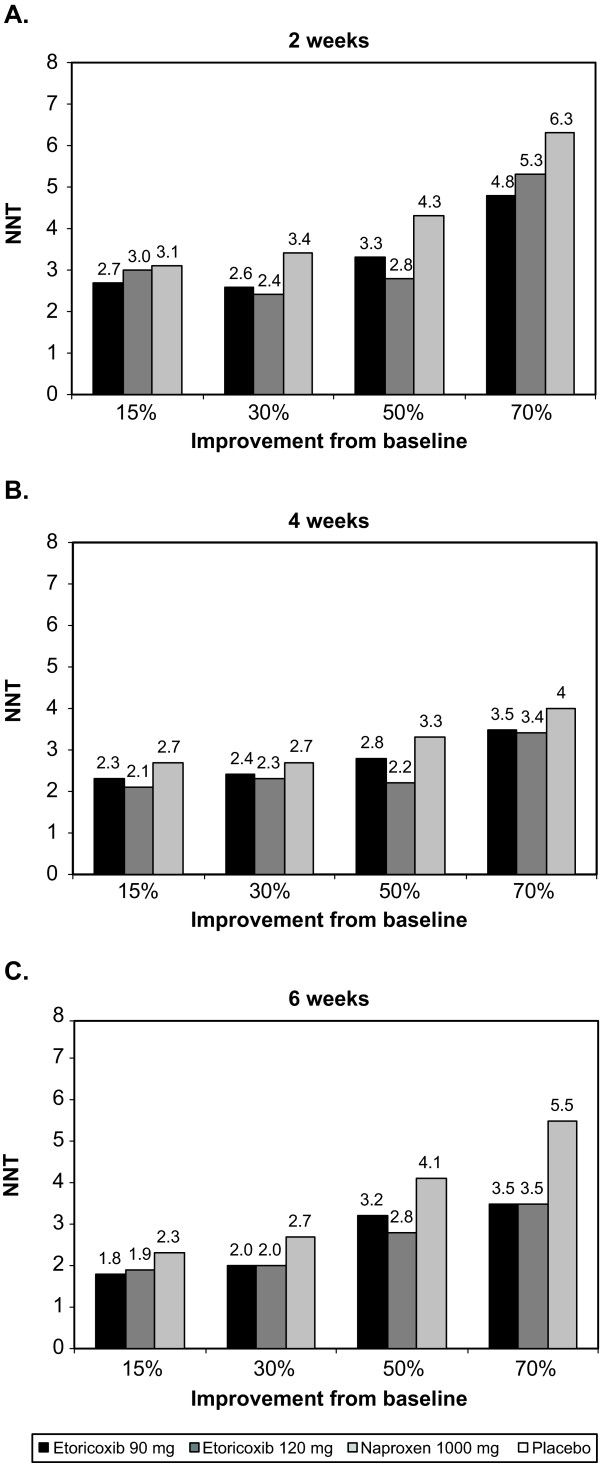
**NNTs vs. placebo for various thresholds of improvement in BASDAI from baseline at (A) 2 weeks, (B) 4 weeks, and (C) 6 weeks**.

**Figure 5:**
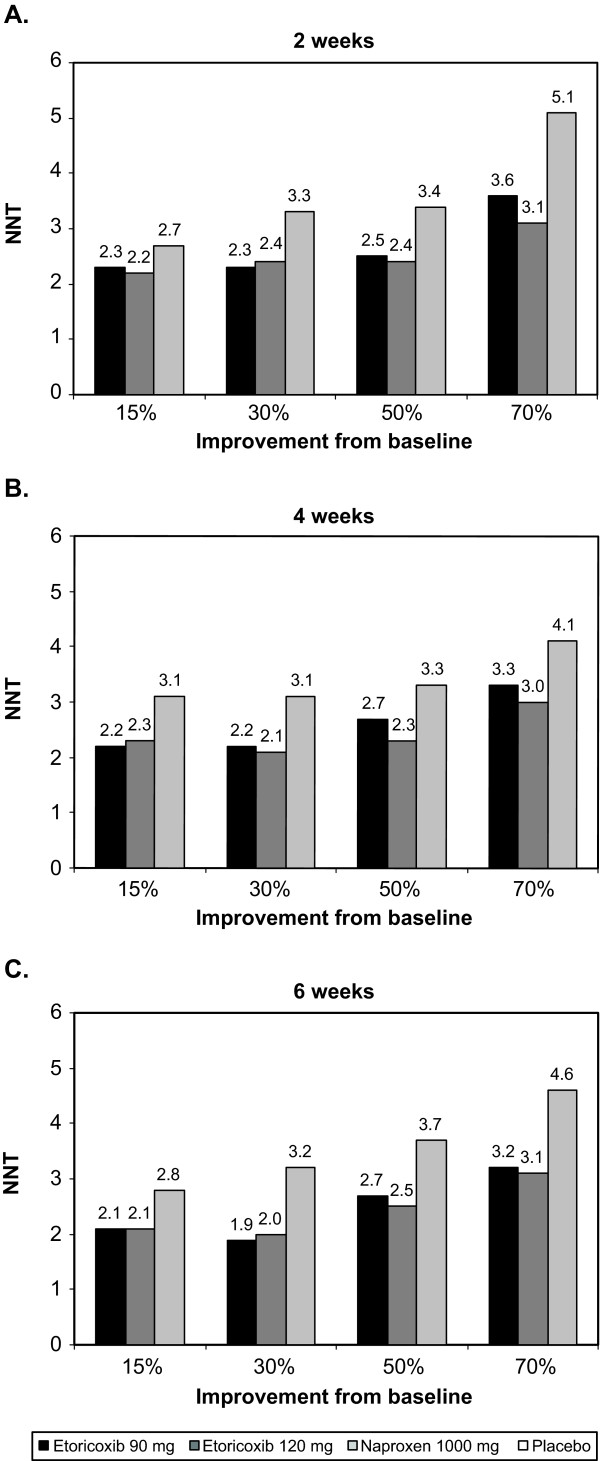
**NNTs vs. placebo for various thresholds of improvement in spine pain from baseline at (A) 2 weeks, (B) 4 weeks, and (C) 6 weeks**.

Consistent with the results for the percentages of responders, NNTs were higher (i.e., worse) when higher thresholds of response were required for both BASDAI (Figure 4) and spine pain (Figure 5). NNTs generally decreased (i.e., improved) slightly over time, and in all cases were lower (i.e., better) with etoricoxib than with naproxen for both BASDAI (Figure 4) and spine pain (Figure 5). For the outcome of ≥30% improvement, NNTs compared with placebo at 6 weeks were 2.0 or lower for etoricoxib 90 mg or 120 mg, and 2.7 or higher with naproxen 1000 mg with both outcomes. For the outcome of ≥50% improvement, NNTs compared with placebo at 6 weeks were 3.2 or lower for etoricoxib 90 mg or 120 mg, and 3.7 or higher with naproxen 1000 mg.

In most cases for both spine pain and BASDAI score, the NNTs for etoricoxib were at least 0.5 units below those for naproxen, suggesting a clinically relevant difference favoring etoricoxib.

## Discussion

The purpose of this study was to perform a responder analysis and calculate the associated NNTs to examine the influence of response thresholds and the time point chosen for analysis, in a trial comparing etoricoxib, naproxen, and placebo in AS [12]. It was demonstrated that response on the PGART does not follow a normal distribution in AS, but rather, has a highly skewed distribution. These results suggest that group mean results should not be inferred to represent a normal distribution of responses. The implication in the present analysis suggests that group mean results do not reflect responses that patients might expect to achieve, consistent with previous research in other pain states [1-6].

The primary report of this trial showed average improvement in BASDAI at 6 weeks of approximately 50% for the etoricoxib groups and 44% for the naproxen group. By examining the percentage of patients achieving various levels of improvements ranging from 15 to 70%, we found that at week 6, 70% of patients receiving etoricoxib and 57% of patients receiving naproxen experienced moderate improvement (≥30%) in BASDAI, and that approximately 48% and 38% experienced substantial improvement (≥50%), with 30% and 20% experiencing extensive (≥70%) improvement. Further, the percentages for spine pain response were generally similar. This provides a very different picture of response than mean changes, and would seem more relevant in communicating treatment expectations with patients. Consistent with the original report and the group mean analysis, substantially more patients receiving etoricoxib or naproxen achieved various thresholds of response for both BASDAI and spine pain than did patients receiving placebo, where active treatments were significantly more effective than placebo for both measures (p < 0.001) [12].

The percentages of responders at 6 weeks for a given threshold were numerically higher for etoricoxib than naproxen, by about 10% for both BASDAI and spine pain. These differences favoring etoricoxib are reflected in the lower (better) NNTs with etoricoxib: after 6 weeks, approximately 1 in 2 patients receiving etoricoxib will achieve a 30% improvement beyond that seen with placebo, 1 in 3 will achieve a 50% improvement, and 1 in 4 will achieve a 70% improvement. By contrast, the corresponding values for naproxen are approximately 1 in 3, 1 in 4, and 1 in 6, respectively.

Although a difference of 1 or 2 NNT units may not appear to be consequential, a difference of ± 0.5 NNT units has been previously cited as a meaningful difference [7,15]. Such meaningful differences in NNTs between etoricoxib and naproxen are consistent with the results from the main analysis, in which changes from baseline in BASDAI and spine pain were significantly greater with etoricoxib than naproxen (p < 0.05) [12].

Some important patterns emerged. As noted, the percentages of responders in the active treatment groups were generally stable over time, whereas the percentages of responders in the placebo group tended to decrease over time. This likely reflects the extent and duration that placebo can exert a positive effect in a highly inflammatory condition. This observation likely accounts for the decreasing (improving) NNTs for active treatment over time despite the generally stable percentages of responders. This is interesting, and confirms that effective therapies generate stable, low NNTs in AS, as has been demonstrated in osteoarthritis [5]. Where therapies are less efficacious, NNTs can increase substantially with duration of observation, as has been seen with ibuprofen in osteoarthritis [5], and with pregabalin in fibromyalgia [6].

NNTs in the range 2-3 at ≥30% improvement and 3-4 at ≥50% improvement for etoricoxib and naproxen for AS were much lower than the NNT of 7 seen for etoricoxib 60 mg or 90 mg after 12 weeks about in chronic low back pain [4], and 4-5 for etoricoxib 30 mg or 60 mg or naproxen 1000 mg in osteoarthritis after 12 weeks [5]. In acute postoperative pain, the NNT for ≥50% pain relief over 6 hours was 1.9 compared with placebo [16]. This speaks to the variability of response to both placebo and etoricoxib across various acute and chronic pain conditions.

Group mean results are usually a mandatory requirement for regulatory approvals and, despite the limitations described here, are valuable in demonstrating that a therapy works in general. In addition, statistics using group means are the most efficient clinical trial designs relative to a comparison of proportions. Further, group mean responses are often used by clinicians to make cross-study comparisons among agents, which is often necessary since this is the most common method of reporting in the literature. However, mm-based measurements of population means is likely to be a fairly abstract concept to most patients, and does not inform the individual's likelihood of response. On the other hand, NNTs are more straightforward to understand and likely more easily conveyed to patients. NNTs alone do not present a full efficacy profile, in the same way that group mean results also do not present a full profile, and it would be beneficial to include both types of results in studies to allow a more complete efficacy profile for clinicians accessing the literature. These NNTs have been calculated using outcomes regarded as important both by clinicians [7]and patients [17].

There are two other important aspects to this analysis. First, we looked at response at various timepoints and found that both the percentages of responders in the active treatment groups and the related NNTs were reasonably stable over time, consistent with previous research showing that early response to coxibs is highly predictive of later response [18]. Therefore, clinicians can not only inform their patients as to the degree of response that can be expected, but also the time frame for such responses. Second, we assigned patient discontinuations 0% improvement from the point of dropout forward through the remainder of the study [19]. By contrast, analyses often use the "last observation carried forward" (LOCF) approach, which can lead to an inflated efficacy result. For example, a patient who experienced good efficacy but who discontinued after week 2 because of an adverse experience would have his or her favorable efficacy score carried forward for another 4 weeks despite not receiving treatment during that time. Our more conservative approach of assigning such patients as nonresponders from that point onward is a more accurate assessment of efficacy and tolerability, and more representative of real-world benefit/risk.

The main limitation of this analysis is that it was post-hoc, although we did use established definitions for meaningful response [7]. Because of its descriptive nature, no formal statistical testing was performed, and results should be viewed as hypothesis-generating. These findings are nonetheless consistent with the primary analysis, with respect to the relative efficacies of etoricoxib, naproxen, and placebo [12]. Since this was an analysis of a single AS trial of just 6 weeks' duration, and with smaller patient numbers compared with responder analyses meta-analyses [5,6] even though there were comparable to numbers studies in CLBP [4], these results should be replicated in other AS datasets. Whether the results demonstrated for etoricoxib and naproxen can be extrapolated to other NSAIDs or to other analgesics used to treat AS, or whether these results apply beyond the 6 week timepoint of the analysis cannot be addressed in this dataset, and remains unknown. However, we note that Dougados and colleagues found that the percentage of responders to piroxicam and meloxicam in AS was similar at 6 weeks and 1 year [20], and Reginster and colleagues found that response to etoricoxib and naproxen had similar efficacy in OA through 138 weeks, suggesting response to NSAIDs is fairly stable [21]. Interestingly, however, in an analysis of data from 7 OA trials, Moore and colleagues found that both the percentages of responders and NNTs were generally stable over 12 weeks for etoricoxib, celecoxib and naproxen, but were relatively unstable for ibuprofen, particular for the NNTs, which increased (worsened) by as much as nearly 200% [5].

## Conclusions

In conclusion, we found that individual patient response is not normally distributed, and rather tends to fall toward the extremes, and as such is not accurately represented by group mean responses. Group mean data are important for understanding that a drug is clearly superior to placebo, but are less valuable for informing patients of expected responses. Responder analyses and NNTs should be presented alongside group mean clinical trial results as they provide a complementary view of efficacy data facilitate doctor-patient discussions of expected benefits, and are more clinically relevant.

## Competing interests

The original study and the present analysis were sponsored by Merck & Co., Inc. Drs. Peloso, Smugar, and Gammaitoni are employees of Merck, and may own or have options to own company stock. Dr. Wang was an employee of Merck when the analysis was performed. Dr. Moore has received research grants, consulting, or lecture fees from pharmaceutical companies, including Pfizer, Merck, GlaxoSmithKline, AstraZeneca, and Grünenthal, but received no remuneration for this work.

## Authors' contributions

HW performed the statistical analysis. SSS wrote the first draft. All authors analyzed and interpreted the data, reviewed and critically revised the initial draft, and all authors approved of the final submitted manuscript. No outside writing assistance was provided.

## Pre-publication history

The pre-publication history for this paper can be accessed here:

http://www.biomedcentral.com/1471-2474/12/165/prepub
